# Probiotic Fermentation Enhances Anti-Diabetic Effects of *Chlorella pyrenoidosa* in Mice by Modulating Gut Microbiota and Short-Chain Fatty Acids

**DOI:** 10.3390/foods15101739

**Published:** 2026-05-14

**Authors:** Juntao Huang, Yue Zhong, Yang Wu, Siqiao Cai, Wenyu Xiong, Tiantian Li, Bin Liu, Zirui Huang

**Affiliations:** 1 College of Food Science, Fujian Agriculture and Forestry University, Fuzhou 350002, China; 2 Engineering Research Centre of Fujian-Taiwan Special Marine Food Processing and Nutrition, Ministry of Education, Fuzhou 350002, China; 3 National Engineering Research Center of JUNCAO Technology, Fuzhou 350002, China; 4 School of Food Science and Engineering, South China University of Technology, Guangzhou 510640, China; 5 Fuzhou Institute of Oceanography, Minjiang University, Fuzhou 350108, China

**Keywords:** *Chlorella pyrenoidosa*, compound probiotics fermentation, anti-diabetic activity, gut microbiota, short-chain fatty acids

## Abstract

Numerous studies have demonstrated that *Chlorella pyrenoidosa* exerts potent anti-diabetic effects, yet its underlying mechanism remains elusive. Notably, no research has explored the anti-diabetic efficacy of its fermented product to date. In this study, we developed a novel compound probiotic-fermented *C. pyrenoidosa* product (CH-F), and systematically evaluated its anti-diabetic efficacy and mechanism. Furthermore, untargeted metabolomics analysis revealed that fermentation significantly altered the metabolite profile of *C. pyrenoidosa*, leading to the enrichment of potential anti-diabetic compounds such as acetylcholine and citicoline. In streptozotocin-induced T2DM mice, CH-F effectively ameliorated hyperglycemia, dyslipidemia, and hepatic/pancreatic histological injuries, showing markedly superior efficacy compared to unfermented *C. pyrenoidosa*. Additionally, CH-F modulated the gut microbiota and improved the levels of short-chain fatty acids (SCFAs) in the intestines of T2DM mice. We demonstrate that CH-F exerts anti-diabetic effects via gut microbiota modulation and SCFA regulation, highlighting its great potential as a functional food ingredient and dietary supplement for the adjuvant management of T2DM.

## 1. Introduction

Diabetes mellitus (DM) is one of the most prominent global public health issues. Epidemiological studies have demonstrated that the number of individuals with DM will increase to 592 million by 2035 [1]. Among individuals with diabetes, type 2 DM (T2DM) accounts for 95% [2]. Despite advances in pharmacotherapeutics, the rising prevalence of diagnosed cases and the economic burden annually underscore the urgent need for safe, adjunctive therapeutic strategies.

Mounting evidence indicates that the gut microbiota acts as a key regulator of metabolic homeostasis, whereas gut dysbiosis is closely linked to impaired glucose metabolism and insulin resistance in patients with T2DM [3]. Probiotics have demonstrated considerable capacity to modulate the gut microbiota, produce bioactive metabolites, and alleviate diabetes-related pathophysiological alterations [4]. The genus *Lactobacillus* represents one of the most representative groups of probiotics, with well-documented efficacy in regulating gut homeostasis and ameliorating metabolic disorders [5]. Among them, *Lacticaseibacillus rhamnosus*, *Lactiplantibacillus plantarum*, *Limosilactobacillus reuteri*, *Lactobacillus acidophilus*, and *Lactobacillus johnsonii* (the former three were previously classified under the genus *Lactobacillus*) are representative species, and accumulating studies have verified their hypoglycemic, hypolipidemic or metabolic regulatory potential [6,7]. However, the efficacy of single-strain probiotics is often limited due to the complexity of the gut ecosystem, and accumulating evidence suggests that multi-strain compound probiotics can exert synergistic effects, achieving more stable and pronounced regulatory effects compared with single strains [8].

*Chlorella pyrenoidosa* is a highly valuable unicellular green alga that is rich in various bioactive substances, including polypeptides, polysaccharides, and *Chlorella* Growth Factor, thereby facilitating extensive applications in the fields of food and pharmaceuticals [9]. Notably, accumulating evidence from numerous studies has demonstrated the anti-diabetic properties of *C. pyrenoidosa* extracts in recent years [10,11]. Additionally, in our previous studies, we further demonstrated that *C. pyrenoidosa* functional formulations exert anti-diabetic effects in T2DM mice by modulating the gut microbiota [12,13].

However, existing studies focused solely on the anti-diabetic effects of *C. pyrenoidosa* extracts or formulations, and did not explore the potential enhancement of its anti-diabetic efficacy through probiotic fermentation. To fill this research gap, the present study aims to develop a novel compound probiotic (CP)-fermented *C. pyrenoidosa* product (CH-F), systematically evaluate its anti-diabetic efficacy, and investigate the underlying regulatory mechanism associated with gut microbiota and short-chain fatty acids (SCFAs), to provide a theoretical basis for the development of algae-derived hypoglycemic functional foods.

## 2. Materials and Methods

### 2.1. Materials and Reagents

*C. pyrenoidosa* was purchased from Xindaze Spirulina Co., Ltd., Fuqing City, China. *L. rhamnosus* (strain no.: ATCC 53013), *L. plantarum* (strain no.: ATCC 14917), *L. acidophilus* (strain no.: ATCC 4796), *L. johnsonii* (strain no.: ATCC 43121), and *L. reuteri* (strain no.: ATCC 53608) were obtained from the China Center of Industrial Culture Collection (Beijing, China). The main reagents used in this experiment, including salicylic acid, ferrous sulfate, absolute ethanol, anhydrous glucose, potassium persulfate, potassium ferricyanide, hydrogen peroxide (H_2_O_2_), ferric chloride, trichloroacetic acid (TCA), sodium carbonate (Na_2_CO_3_), sulfuric acid, and gallic acid, were all purchased from Sinopharm Chemical Reagent Co., Ltd. (Shanghai, China). All assay kits utilized in this study were procured from Nanjing Jiancheng Bioengineering Institute.

### 2.2. Revival of ATCC Strains

The bacterial suspension was inoculated into MRS medium at an inoculum size of 2%, followed by incubation at a constant temperature of 37 °C for 24 h. Subsequently, the probiotic fermentation broth was centrifuged at 4000 rpm for 10 min. The obtained probiotic cell precipitate was resuspended 2–3 times with 0.9% normal saline, and then normal saline was added to adjust the cell concentration to approximately 10^9^ CFU/mL to prepare the bacterial suspension for subsequent use. The supernatant was collected by filtering the centrifuged supernatant through a 0.22 μm sterile filter membrane and stored at 4 °C. In addition, the original fermentation broth was also adjusted to a cell concentration of about 10^9^ CFU/mL with normal saline.

The activated strains were individually inoculated into 5 mL of MRS medium and incubated at 37 °C. Every 2 h, 3 tubes were sampled to determine the optical density (OD) at 600 nm, pH value and CFU count, with the whole assay conducted over a total duration of 48 h.

### 2.3. Determination of Antioxidant Activity In Vitro

To evaluate the in vitro antioxidant activity of candidate strains and subsequent compound probiotic formulations, we adopted the following standardized assays. The DPPH assay was performed with slight modifications based on the previous method [14]. A 0.2 mM DPPH solution was prepared and stored protected from light. One hundred microliters of the sample was mixed with an equal volume of the DPPH solution, and the mixture was reacted at room temperature in the dark for 30 min prior to absorbance measurement at 517 nm. For the control group, the DPPH solution was substituted with absolute ethanol of the same volume; for the blank group, the sample was replaced with absolute ethanol.

$$\;DPPH\;radical\;scavenging\;activity\;(\%)=\left[1-\frac{\left(A_{1}{-A}_{2}\right)}{A_{0}}\right]\times 100\%$$

where A_1_ = absorbance of sample group; A_2_ = absorbance of control group; and A_0_ = absorbance of blank group.

The ABTS free radical scavenging activity was determined with slight modifications based on the reported method [15]. Briefly, the ABTS working solution was prepared by mixing 7.4 mM ABTS with 2.6 mM potassium persulfate at 1:1, incubated at room temperature in the dark. It was then diluted 40-fold with 1 × PBS to an absorbance of 0.7 ± 0.02 at 734 nm. Subsequently, 160 μL ABTS working solution was mixed with 40 μL of the sample, incubated at 30 °C for 6 min, and absorbance measured at 734 nm. Control group: ABTS working solution replaced with 1 × PBS. Blank group: Sample replaced with 1 × PBS. The scavenging activity was calculated using the formula for DPPH radical scavenging activity.

The OH radical scavenging activity was also determined with reference to the previous report [15]. A 6.7 mM H_2_O_2_ solution was prepared. Subsequently, 500 μL of the sample was mixed with 175 μL of H_2_O_2_, and incubated at 37 °C for 12 min, followed by the addition of 75 μL of salicylic acid. After further incubation at 37 °C for 30 min, the absorbance was measured at 562 nm. For the control group, salicylic acid was replaced with ultrapure water; for the blank group, the sample was replaced with ultrapure water. The scavenging activity was calculated using the formula for DPPH radical scavenging activity.

Five hundred microliters of the sample were mixed with 100 μL of PBS (0.2 mol/L, pH 6.6) and 250 μL of 1% K_3_[Fe(CN)_6_]. After thorough mixing, the mixture was incubated in a 50 °C water bath for 20 min. Following cooling in an ice-water bath, 500 μL of 10% TCA was immediately added and vortexed well. Subsequently, 100 μL of 1% FeCl_3_ was introduced and mixed uniformly. After reacting at room temperature for 5 min, the absorbance was measured at 700 nm. For the control group, K_3_[Fe(CN)_6_] was replaced with deionized water; for the blank group, the sample was replaced with deionized water.

$${The\;ferric\;reducing\;antioxidant\;power=A}_{1}{-A}_{2}{-A}_{0}$$

where A_1_ = absorbance of sample group; A_2_ = absorbance of control group; and A_0_ = absorbance of blank group.

### 2.4. Determination of Hypoglycemic Inhibitory Activities In Vitro

To evaluate the in vitro hypoglycemic inhibitory activities of candidate strains and subsequent compound probiotic formulations, we adopted the following standardized assays. The method for determining the inhibitory activity of α-glucosidase refers to our previous study [16]. A 30 μL sample was mixed with 60 μL PBS (pH 6.8), then 30 μL α-glucosidase (from *Saccharomyces cerevisiae*, ≥50 U/mg; Shanghai Yuanye Bio-Technology Co., Ltd., Shanghai, China) solution (0.2 U/mL) and 30 μL PNPG solution (5 mmol/L) were added; the mixture was incubated at 37 °C for 20 min, and the reaction was terminated with 50 μL of 0.1 mol/L Na_2_CO_3_ solution, followed by absorbance measurement at 405 nm. For the sample control, α-glucosidase was replaced with 1 × PBS; for the positive control, the sample was replaced with 1 × PBS; for the blank control, both the sample and α-glucosidase were replaced with 1 × PBS. The inhibition rate was calculated as:

$$\alpha \text{-}glucosidase\;inhibitory\;activity\;(\%)=\left[1-\frac{\left(A_{1}{-A}_{2}\right)}{\left({A_{3}-A}_{0}\right)}\right]\times 100\%$$

where A_1_ = absorbance of sample group, A_2_ = sample control, A_3_ = positive control, and A_0_ = blank control.

For α-amylase inhibitory activity, 1 mg/mL α-amylase solution was prepared, and 1.5% (*w*/*v*) soluble starch solution was preheated to 37 °C. A total of 500 μL α-amylase (from porcine pancreas, ≥10 U/mg; Beijing Solarbio Science & Technology Co., Ltd., Beijing, China) solution was added to the sample and incubated at 37 °C for 10 min, then 1 mL preheated starch solution was supplemented and incubated for another 5 min at 37 °C. The reaction was terminated with 1 mL DNS reagent, heated in a boiling water bath for 5 min, cooled in an ice-water bath for 5 min, and left to stand at room temperature for 20 min before measuring absorbance at 540 nm. The sample control, positive control and blank control were set by the same substitution method with 1 × PBS as above, and the inhibition rate was calculated using the same formula as α-glucosidase.

### 2.5. Formulation of Probiotics

Based on the results of the hypoglycemic and antioxidant activity assays in vitro, *L. rhamnosus*, *L. plantarum*, and *L. acidophilus* were combined as single-strain, dual-strain, and triple-strain formulations, yielding a total of 7 probiotic combinations (Table 1). Each combination was inoculated into the *C. pyrenoidosa* medium (CHM) at an inoculum size of 2% (*v*/*v*) for fermentation. The CHM was formulated with 3% (*w*/*v*) *C. pyrenoidosa* powder and 3% (*w*/*v*) glucose. The components were dissolved in distilled water, the pH was adjusted to 6.8, and the mixture was vortexed for 2 min, then autoclaved and cooled to obtain the final medium, with fermentation performed at 37 °C for 24 h. The hypoglycemic activity in vitro, pH value, and viable cell count (CFU) of the 24 h fermentation products from the 7 combinations were used as evaluation indicators to screen for probiotic formulations with superior hypoglycemic activity and growth performance.

### 2.6. Process Optimization of Compound Probiotic-Fermented C. pyrenoidosa

To optimize the fermentation process of CP-fermented *C. pyrenoidosa* solution (CH), five single factors were investigated and optimized, which were as follows: glucose content (1%, 2%, 3%, 4%, and 5%), *C. pyrenoidosa* content (2%, 3%, 4%, 5%, and 6%), probiotic inoculum size (1%, 2%, 3%, 4%, and 5%), fermentation temperature (31 °C, 34 °C, 37 °C, 40 °C, and 43 °C), and fermentation time (12 h, 18 h, 24 h, 30 h, and 36 h). Factors that exerted significant effects on the hypoglycemic activity, pH value, and CFU count of the fermentation products in the single-factor experiments were selected for orthogonal optimization experiments. A four-factor and three-level orthogonal experiment was established, and the specific design of experimental factors and levels is presented in Table 2. The remaining factors were set at their optimal levels derived from the single-factor experiments. Taking the hypoglycemic activity, pH value, and CFU count as the evaluation indices, the experimental results were comprehensively assessed by the Z-score comprehensive evaluation method, as detailed in the following equations.

$$Z_{i}=\frac{\left(Y_{i}-\bar{Y}i\right)}{S_{i}}\;\sum Z_{i}=\sum Z_{i,HB}-\sum Z_{i,LB}$$

where Z_i_ = Z-score of each target indicator, Y_i_ = measured value of the specific indicator, $\bar{Y}$_i_ = mean value of the corresponding indicator, S_i_ = standard deviation of the corresponding indicator, ∑Z_i_ = total comprehensive Z-score of a test group, ∑Z_i, HB_ = sum of Z-scores for indicators where larger values represent better experimental performance within the test group, and ∑Z_i, LB_ = sum of Z-scores for indicators where smaller values represent better experimental performance within the test group.

### 2.7. Preparation of Samples

**Figure 1:**
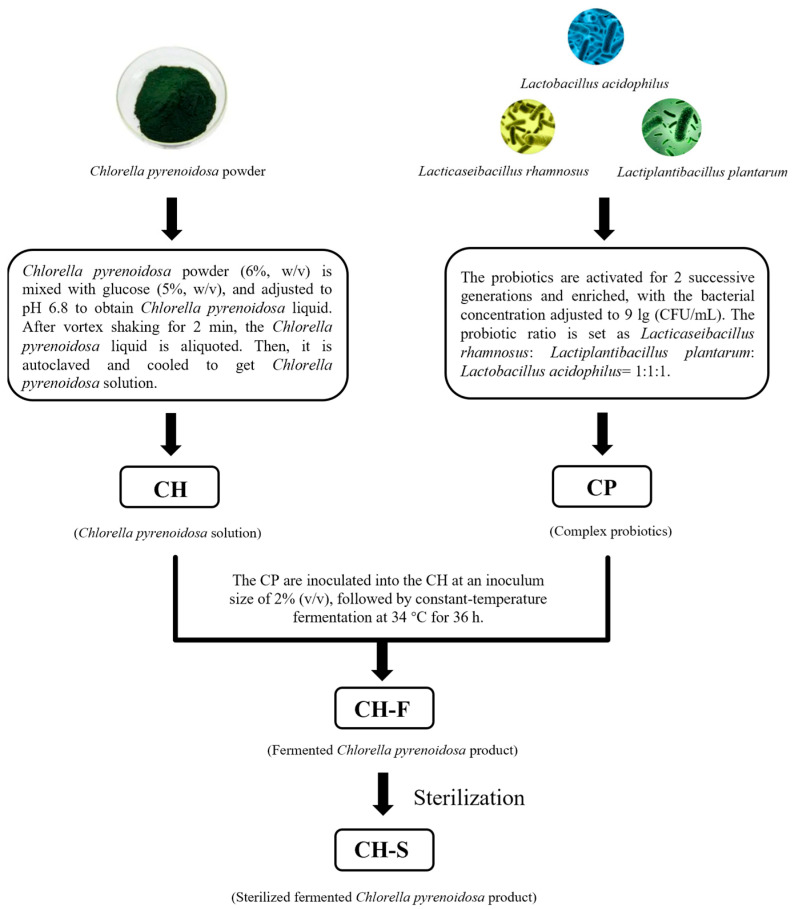
The preparation process of CH, CP, CH-F and CH-S.

Based on the results of single-factor experiments and orthogonal optimization of the preparation process, CH, CP, CH-F and sterilized fermented *C. pyrenoidosa* product (CH-S) were subsequently prepared, with the detailed procedures and process parameters illustrated in Figure 1.

### 2.8. Determination of Nutritional Component Contents

The content of crude polysaccharides was determined by the phenol–sulfuric acid method, following a previously reported protocol [17]. Briefly, 500 μL of each sample was mixed with an equal volume of 5% phenol solution, followed by slow addition of 2 mL concentrated sulfuric acid along the test tube wall. The mixture was gently shaken to homogenize, incubated in a boiling water bath for 15 min and cooled to room temperature, then its OD_490_ was measured. A glucose-based standard curve was established, and the absorbance values were fitted to the curve for the calculation of crude polysaccharide content in the samples.

The content of reducing sugars was determined by the dinitrosalicylic acid (DNS) method, following a previously reported protocol [18]. Briefly, 100 μL of each sample was mixed with an equal volume of DNS reagent, and the mixture was heated in a boiling water bath for 5 min, immediately cooled in an ice-water bath for 5 min and then 20-fold diluted, followed by OD_540_ measurement. A glucose standard curve was established, and the sample reducing sugar content was calculated by interpolating the absorbance values into this curve.

The content of total phenols was determined by the Folin–Ciocalteu method. Briefly, 40 μL of each sample was added to a 96-well microplate, and an equal volume of Folin–Ciocalteu reagent was supplemented. The mixture was incubated at 50 °C for 30 min for color development, and the reaction was terminated by adding 120 μL of 20% (*w*/*v*) Na_2_CO_3_ solution, followed by absorbance measurement at 765 nm. A gallic acid-based standard curve was constructed, and the sample total phenol content was calculated by interpolating the absorbance values into the curve.

The content of proteins was determined by the bicinchoninic acid method. Briefly, 2 mL of working solution (Solution A: Solution B = 50:1, *v*/*v*) was added to 100 μL of each sample, well mixed, and incubated at 37 °C for 30 min, followed by absorbance measurement at 562 nm. A standard curve was constructed using bovine serum albumin (BSA) as the reference, and the sample protein content was calculated by interpolating the absorbance values into this curve.

### 2.9. Identification of Metabolites by UPLC-MS/MS

To investigate the effect of probiotic fermentation on the metabolite profile of *C. pyrenoidosa*, we performed untargeted metabolomics analysis on both unfermented CH and CH-F using UPLC-MS/MS. Briefly, 100 μL of each liquid sample was pipetted into EP tubes, and 500 μL of 80% methanol aqueous solution was added. After vortexing, the mixture was incubated in an ice-water bath for 5 min and centrifuged at 15,000 rpm and 4 °C for 20 min. The supernatant was collected, mixed with 53% methanol aqueous solution, and centrifuged again under the same conditions. The resulting supernatant was filtered through a 0.22 μm membrane to obtain the sample for UPLC-MS/MS analysis. Chromatographic conditions included a Hyperil Gold C18 column, with the column temperature maintained at 40 °C, a flow rate of 0.2 mL/min, mobile phase A as 0.1% formic acid and mobile phase B as methanol. Data were processed using the Compound Discoverer 3.3 (CD3.3) software for spectrum analysis and database searching. The mass-to-charge ratio (*m*/*z*), retention time and peak area were acquired; molecular weight was calibrated with a mass deviation < 5 ppm. Metabolite annotation was confirmed by matching MS/MS fragmentation patterns with the HMDB (https://hmdb.ca/metabolites) (accessed on 7 September 2023).

### 2.10. Animal Experiment

A total of 42 male specific pathogen-free (SPF) ICR mice (4 weeks old, 26 ± 2 g) were provided by the Wushi Experimental Center Laboratory (Fuzhou, China). The mice were housed in environmentally controlled rooms, with a temperature of 25 ± 5 °C, relative humidity of 50 ± 10%, and a 12/12 h light/dark cycle. After the acclimatization period, 6 mice were randomly assigned to the normal control (NC) group and continued to be fed a maintenance diet. After fasting for 8–12 h, the remaining 36 mice were intraperitoneally injected with streptozotocin (STZ) solution at a dose of 125 mg/kg, and were fed a high-sugar and high-fat diet within the following week. One week later, the mice were fasted for 8–12 h, and their fasting blood glucose (FBG) was measured. Subsequently, these 36 mice were randomly divided into the following 6 groups: the model control (MC) group, metformin hydrochloride (MH) group, CH group, CH-F group, CH-S group, and CP group.

### 2.11. Sampling and Determination

Following successful establishment of the mouse model, the FBG and body weight (BW) of the mice were recorded at a fixed time point every week. After 4 weeks of intragastric administration, mice were fasted for 12 h with free access to water prior to the FBG measurement and oral glucose tolerance test (OGTT). Mice were intragastrically administered with glucose solution at a dose of 2 g/kg body weight, and the blood glucose levels of each mouse were measured at 0 h, 0.5 h, 1 h and 2 h after glucose administration. The area under the curve (AUC) for the OGTT was calculated using the following formula. Subsequently, all mice were fasted for 12 h with ad libitum access to water and euthanized by cervical dislocation. Blood samples were promptly collected from the retro-orbital venous plexus immediately. After 30 min, the blood samples were centrifuged to collect the supernatant, which was stored at −80 °C. Mice were then sacrificed by cervical dislocation, and the liver, pancreas and intestinal contents were harvested via dissection and temporarily stored at −20 °C. Partial liver tissues were fixed in 4% paraformaldehyde solution and kept at room temperature, while the remaining tissues were snap-frozen in liquid nitrogen and stored at −80 °C.

The total cholesterol (TC) and triglyceride (TG) levels in mice livers were determined using corresponding commercial assay kits. Meanwhile, the TC, TG, glycosylated serum protein (GSP), superoxide dismutase (SOD), malondialdehyde (MDA), and serum insulin (INS) in the serum of mice were measured. Additionally, the insulin sensitivity index (HOMA-ISI), insulin resistance index (HOMA-IRI), and pancreatic β-cell function index (HOMA-β) were calculated using the following formulas.

$$AUC=\left(G_{0}+G_{0.5}\right)\times 0.25+\left(G_{0.5}+G_{1.0}\right)\times 0.25+\left(G_{1.0}+G_{2.0}\right)\times 0.5$$

$$HOMA\text{-}IRI=\left(\frac{FBG\times INS}{22.5}\right)\times 100\%$$

$$HOMA\text{-}ISI=\left(\frac{22.5}{FBG\times INS}\right)\times 100\%$$

### 2.12. Assessment of Histopathology

Liver and pancreatic tissues were fixed, paraffin-embedded, sectioned, and stained with hematoxylin and eosin. The morphological features of the sections were observed under a light microscope [19].

### 2.13. Analysis of Gut Microbiota

16S rRNA sequencing was performed at Shanghai Lingen Biotechnology Co., Ltd. (Shanghai, China), and the specific methods were described in our previous study [20]. The alpha diversity of gut microbiota was analyzed using the R software (v4.0.2). Linear discriminant analysis effect size (LEfSe) analysis was performed, with the linear discriminant analysis (LDA) score threshold set at > 3. Functional prediction of the mouse gut microbiota was conducted via PICRUSt2. The Spearman correlation test was applied to assess the associations between the gut microbiota, SCFAs and biochemical indicators, and the correlation results were visualized using Cytoscape (v3.7.1).

### 2.14. Statistics

All experimental data are presented as mean ± standard deviation (SD). One-way analysis of variance (ANOVA) was performed using GraphPad Prism (Version 9.0), followed by Tukey’s test for post-hoc multiple comparisons. Significant differences among groups are indicated by different lowercase letters (a, b, c, and d), while the same letter represents no significant difference at the level of *p* < 0.05.

## 3. Results

### 3.1. Screening Results of Hypoglycemic Probiotics

**Figure 2:**
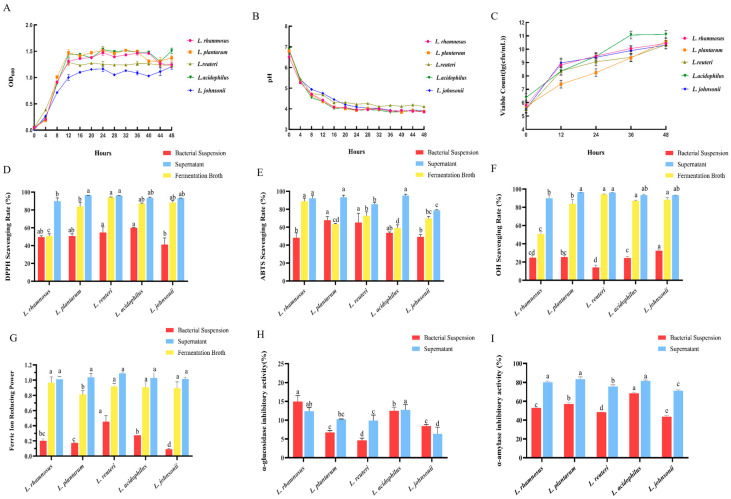
Screening and characterization of five probiotic strains in vitro (*n* = 3). The optical density at 600 nm (**A**), pH (**B**), and CFU counts (**C**) in the culture medium of five probiotic strains during 48 h. In vitro antioxidant and hypoglycemic activities of five probiotic strains: DPPH (**D**), ABTS (**E**), OH (**F**) radical scavenging activities, ferric reducing antioxidant power (**G**), α-glucosidase (**H**) and α-amylase (**I**) inhibitory activities. All data are presented as mean ± SD. One-way ANOVA followed by Tukey’s post-hoc test was used for group comparisons. Different lowercase letters (a,b,c,d) indicate significant differences at *p* < 0.05, while the same letter represents no significant difference.

To identify the optimal candidate strains for subsequent compound fermentation, we first evaluated the growth characteristics, acid production capacity, viable counts, and hypoglycemia-related in vitro activities of five candidate *Lactobacillus* strains over 48 h of cultivation. All strains showed favorable probiotic growth characteristics, steady pH decline and high viability, with *L. acidophilus* having the highest CFU counts, followed by *L. plantarum* and *L. rhamnosus* (Figure 2A–C). As is shown in Figure 2D–G, all strains exhibited robust antioxidant potential, and each strain’s fermentation supernatant had significantly stronger antioxidant activity than the corresponding bacterial suspensions and whole fermentation broths. As shown in Figure 2H, *L. rhamnosus* and *L. acidophilus* exhibited the highest α-glucosidase inhibitory activity. As shown in Figure 2I, the supernatants of all strains exerted stronger inhibitory effects on α-amylase than their corresponding bacterial suspensions. Among these, the α-amylase inhibitory rates of *L. rhamnosus*, *L. plantarum*, and *L. acidophilus* were significantly higher than those of the other two strains (*p* < 0.05). Based on a comprehensive evaluation of all the above indicators, *L. plantarum*, *L. acidophilus*, and *L. rhamnosus* exhibited the best overall performance.

### 3.2. Optimization Results of Probiotic Formulation

**Figure 3:**
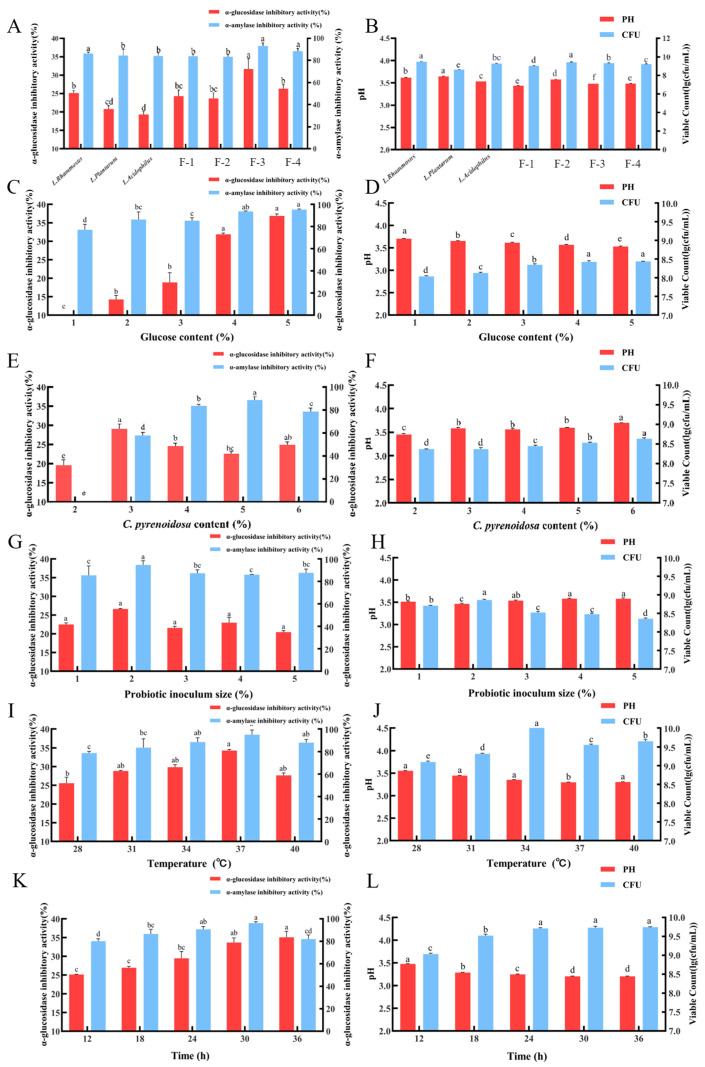
Analysis on the formulation of probiotics and single-factor optimization of the fermentation process (*n* = 3). In vitro hypoglycemic activities (**A**), pH and viable cell counts (**B**) of three probiotic strains and four compound formulations. Effects of glucose content, *C. pyrenoidosa* content, probiotic inoculum size, fermentation temperature and time on in vitro hypoglycemic activities and pH and CFU counts of fermentation supernatant (**C**–**L**). All data are presented as mean ± SD. One-way ANOVA followed by Tukey’s post-hoc test was used for group comparisons. Different lowercase letters (a,b,c,d) indicate significant differences at *p* < 0.05, while the same letter represents no significant difference. Abbreviations: F-1–F-4, presented in subfigures (**A**,**B**), denote the four compound probiotic formulations with strain ratios and compositions detailed in Table 1.

To test this hypothesis and determine the optimal combination, we formulated the three selected strains into seven single-strain, dual-strain and triple-strain combinations (Table 1), and compared their in vitro hypoglycemic activities, pH values, and viable counts across seven formulations. As illustrated in Figure 3A, the supernatant from the F-3 group exhibited the highest α-glucosidase inhibition rate at 31.66%, followed by those from the F-4 group and *L. rhamnosus*, with inhibition rates of 26.30% and 25.12%, respectively. Similarly, the highest α-amylase inhibition rate was also detected in the F-3 group at 92.98%, followed by the F-4 group at 88.28%. This 1:1:1 triple-strain combination in the F-3 group exhibited higher in vitro hypoglycemic enzyme inhibitory activity than all single-strain formulations. As shown in Figure 3B, the pH values of the supernatants in the F-1, F-3 and F-4 groups were lower than those of all single-strain groups, whereas the CFU counts of the F-3 group were higher than those of the F-1 and F-4 groups. Based on a comprehensive evaluation of all indicators, the F-3 group was identified as the optimal formulation, with a ratio of *L. rhamnosus*:*L. plantarum*:*L. acidophilus* = 1:1:1.

### 3.3. Optimization Results of Fermentation Process

Figure 3C–L illustrate the effects of glucose content, *C. pyrenoidosa* content, probiotic inoculum size, fermentation temperature and time on the CP-fermented CH. The results indicated that all five factors exerted significant effects on the in vitro hypoglycemic activity, pH value, and viable cell count of the CP-fermented CH product. Based on these findings, *C. pyrenoidosa* contents of 4%, 5%, and 6%, glucose contents of 3%, 4%, and 5%, fermentation temperatures of 34 °C, 37 °C, and 40 °C, and fermentation times of 24 h, 30 h, and 36 h were selected for subsequent orthogonal process optimization, with the probiotic inoculum size fixed at 2%. The orthogonal array test results and corresponding one-way ANOVA results are presented in Table 3 and Table 4, respectively. The Z-score comprehensive evaluation method was used to identify the optimal process, and experimental run 8 showed the highest comprehensive Z-score. The ANOVA results confirmed that factors A (*p* < 0.01), B (*p* < 0.01), C (*p* < 0.01) and D (*p* < 0.05) exerted significant effects on the Z-score. Accordingly, the optimal fermentation process conditions were determined as A_3_B_2_C_1_D_3_, corresponding to a *C. pyrenoidosa* content of 6%, a glucose content of 4%, a fermentation temperature of 34 °C and a fermentation time of 36 h.

### 3.4. Analysis of Constituent Composition and Bioactivities in CH and CH-F

**Figure 4:**
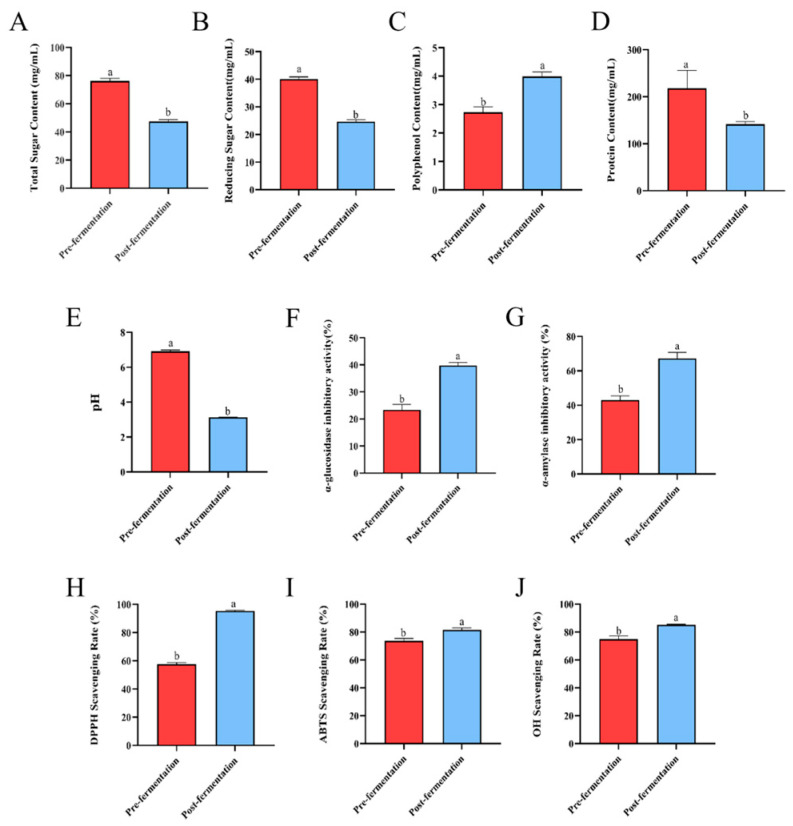
Analysis of constituent composition and bioactivities in CH and CH-F (*n* = 3). Total sugar content (**A**), total reducing sugar content (**B**), total polyphenol content (**C**), total protein content (**D**), and pH (**E**) of CH and CH-F. In vitro α-glucosidase inhibitory activity (**F**) and α-amylase inhibitory activity (**G**), and DPPH (**H**), ABTS (**I**), and OH (**J**) radical scavenging activities of CH and CH-F. All data are presented as mean ± SD. One-way ANOVA followed by Tukey’s post-hoc test was used for group comparisons. Different lowercase letters (a,b) indicate significant differences at *p* < 0.05, while the same letter represents no significant difference.

Figure 4A–D present the total sugar, reducing sugar, polyphenol, and protein content in CH and CH-F. The total sugar (*p* < 0.001), reducing sugar (*p* < 0.001), and protein content (*p* < 0.05) in fermented CH-F were significantly decreased compared with those in CH, while the polyphenol content was significantly increased (*p* < 0.001). Furthermore, the pH value of CH-F was significantly lower than that of CH (Figure 4E). In terms of bioactivity, CH-F exhibited significantly enhanced α-glucosidase and α-amylase inhibitory activities, as well as significantly increased DPPH, OH and ABTS free radical scavenging activities, compared with CH (Figure 4F–J).

### 3.5. Analysis of Metabolites Before and After Fermentation

UPLC-MS/MS analysis was performed (Figure S1), and a comparative analysis between CH and CH-F was conducted using the screening criteria of variable importance in the projection VIP > 1 and *p* < 0.05. A total of 256 differential metabolites were identified. Among these, 85 species were significantly upregulated in CH-F, mainly including organic acids, alkaloids, nucleotides, hydroxy acids and their derivatives; 171 metabolites were significantly downregulated in CH-F, mainly including glycerophospholipids, fatty acyls and organic oxides. Further screening with fold change (FC) > 60 and FC < 0.004 yielded 12 metabolites with the most significant differences, which are presented in Table 5.

### 3.6. CP, CH, CH-F and CH-S Ameliorate Biochemical Indicators in T2DM Mice

#### 3.6.1. Effect of CP, CH, CH-F and CH-S on BW and FBG

**Figure 5:**
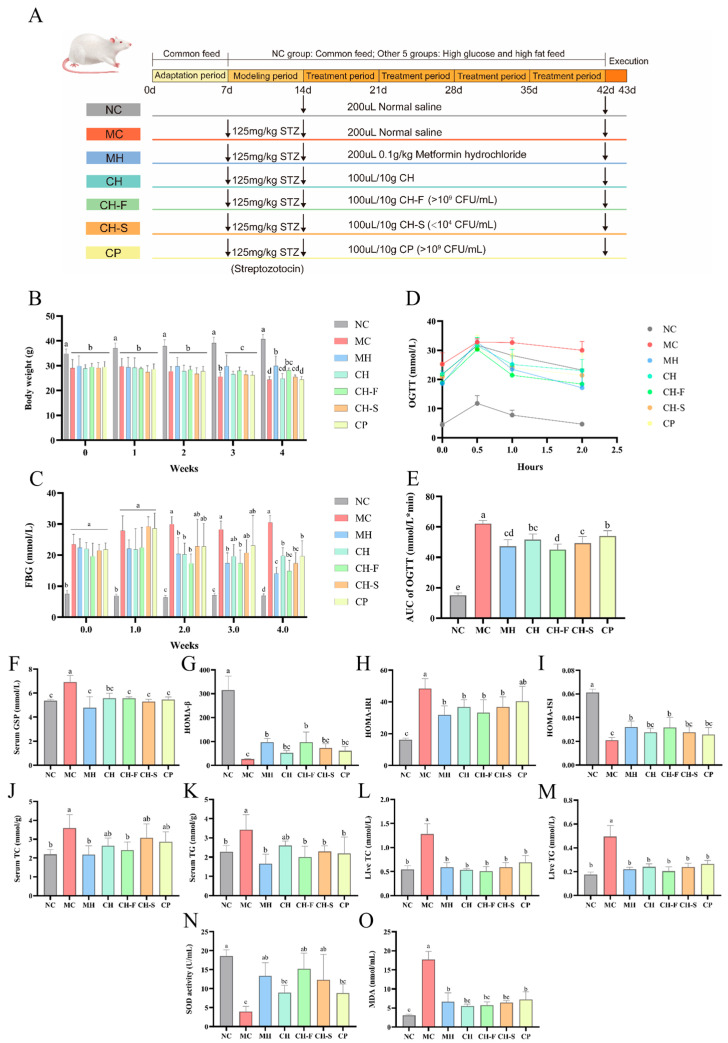
Experimental design and effects of CH, CP, CH-F, and CH-S on biochemical indexes in T2DM mice (*n* = 6). The schematic diagram of the animal experiment (**A**). Effects of CH, CP, CH-F and CH-S administration on the levels of BW (**B**), FBG (**C**), OGTT (**D**), AUC of OGTT (**E**), serum GSP (**F**), HOMA-β (**G**), HOMA-IRI (**H**), HOMA-ISI (**I**), serum TC (**J**), serum TG (**K**), liver TC (**L**), liver TG (**M**), SOD (**N**) and MDA (**O**) in T2DM mice. All data are presented as mean ± SD. One-way ANOVA followed by Tukey’s post-hoc test was used for group comparisons. Different lowercase letters (a,b,c,d) indicate significant differences at *p* < 0.05, while the same letter represents no significant difference. Abbreviations: NC, normal control group; MC, model control group; MH, metformin hydrochloride group; CH, *C. pyrenoidosa* solution group; CH-F, fermented *C. pyrenoidosa* group; CH-S, sterilized fermented *C. pyrenoidosa* group; CP, compound probiotics group.

Mice in each group received intragastric administration for 4 weeks, with the specific administration samples and dosages detailed in Figure 5A. As shown in Figure 5B, at successful model establishment (Week 0), the BW of mice in the NC group was significantly higher than those of mice in all diabetic groups. As the experiment progressed, all groups showed an attenuated weight loss trend by Week 3. After 4 weeks of treatment, the BW levels of mice in the MH and CH-F groups was significantly higher than those in the MC group, with increases of 22.84% (*p* < 0.001) and 15.64% (*p* < 0.05), respectively.

As shown in Figure 5C, after successful modeling, the FBG in all diabetic groups was significantly increased compared with the NC group (*p* < 0.05). After two weeks of treatment, the MH, CH, and CH-F groups significantly reduced the FBG in T2DM mice relative to the MC group (*p* < 0.05). After four weeks, all treatment groups showed a significant reduction in FBG in T2DM mice compared with the MC group. Among these groups, the MH and CH-F groups showed the greatest effects, with reductions of 53.77% (*p* < 0.001) and 51.13% (*p* < 0.001), respectively.

#### 3.6.2. Effect of CP, CH, CH-F and CH-S on OGTT, GSP, and Insulin-Related Indicators

As shown in Figure 5D, the blood glucose levels of mice in all groups increased sharply after glucose administration, peaked at 0.5 h, and then gradually decreased. The MC group had significantly higher blood glucose levels than the NC group at all time points, while all treatment groups showed intermediate blood glucose levels between the NC and MC groups. As shown in Figure 5E, compared with the MC group, the AUC of OGTT was significantly decreased in all treatment groups. Additionally, compared with the NC group, the GSP level was significantly elevated in the MC group (*p* < 0.001), and all treatment groups showed a significant reduction in GSP level (*p* < 0.001) (Figure 5F).

We evaluated the insulin resistance and pancreatic β-cell function in mice using the HOMA-IRI, HOMA-ISI, and HOMA-β indices (Figure 5G–I). Compared with the NC group, mice in the MC group had significantly decreased HOMA-β (*p* < 0.001) and HOMA-ISI (*p* < 0.001), and significantly increased HOMA-IRI (*p* < 0.001). All treatment groups ameliorated these three indices to varying degrees. Among them, only the MH group and CH-F group exhibited a significant increase in HOMA-ISI and HOMA-β compared with the MC group. For HOMA-IRI, the MH group and CH-F group also showed the greatest reduction, with decreases of 34.6% (*p* < 0.01) and 31.4% (*p* < 0.01), respectively, compared with the MC group.

#### 3.6.3. Effect of CP, CH, CH-F and CH-S on Hyperlipidemia

As shown in Figure 5J,K, compared with the MC group, only the MH and CH-F groups significantly decreased the serum TC levels in T2DM mice, with reductions of 39.12% (*p* < 0.001) and 32.79% (*p* < 0.01), respectively. For serum TG, levels were significantly reduced in the CH, CH-F, CH-S, CP and MH groups compared with the MC group, with the MH group showing the greatest reduction, followed by the CH-F group. Meanwhile, compared with the MC group, all treatment groups showed a significant reduction in liver TC and TG levels in T2DM mice, with the CH-F group showing the greatest reduction, with decreases of 60.34% (*p* < 0.001) and 58.78% (*p* < 0.001), respectively (Figure 5L,M).

#### 3.6.4. Effect of CP, CH, CH-F and CH-S on Serum Oxidative Stress

We determined the SOD activities and MDA levels in serum from mice to assess the level of oxidative stress (Figure 5N,O). Compared with the MC group, all treatment groups showed significantly higher serum SOD activity in T2DM mice, with the CH-F group showing the greatest increase of 287.61% (*p* < 0.01). For MDA levels, the CH, CH-F, and CH-S groups showed the most significant reduction, with MDA levels reduced by 68.86% (*p* < 0.001), 67.58% (*p* < 0.001), and 63.47% (*p* < 0.001), respectively, compared with the MC group.

### 3.7. Effects of CP, CH, CH-F and CH-S on the Liver and Pancreas Histopathological Sections

**Figure 6:**
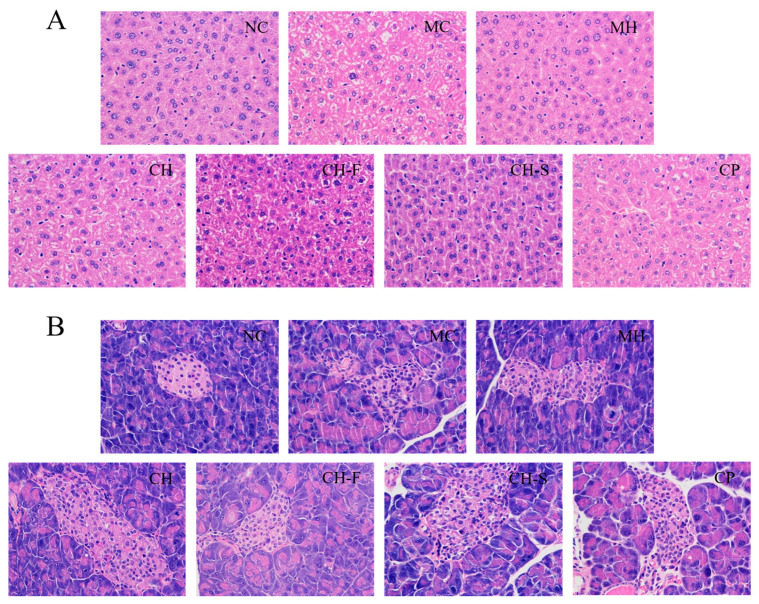
The liver (**A**) and pancreas (**B**) sections by hematoxylin and eosin staining.

Figure 6A,B demonstrates the hematoxylin–eosin stained pathological sections of the liver and pancreas from mice in each group, respectively. In the NC group, the liver and pancreas showed intact histological morphology, with neatly and evenly arranged hepatocytes free of lipid degeneration, as well as pancreatic islets with regular contours and clear boundaries. Compared with the NC group, hepatocytes in the MC group were arranged in a disorganized manner, presenting lipid vacuoles and cellular edema, accompanied by obvious inflammatory infiltration, thereby exhibiting significant hepatic injury. Furthermore, pancreatic tissue in the MC group exhibited marked degeneration, characterized by ill-defined islet margins and structural disorganization, indicating that T2DM exerted damaging effects on the pancreas of mice. Following four weeks of treatment intervention, the hepatic and pancreatic tissue damage in mice from each administration group were improved to varying degrees. Overall, the MH group showed the most obvious improvement in tissue damage, while the CH-F group achieved moderate recovery in both hepatic and pancreatic tissue.

### 3.8. Effects of CP, CH, CH-F and CH-S on Gut Microbiota

**Figure 7:**
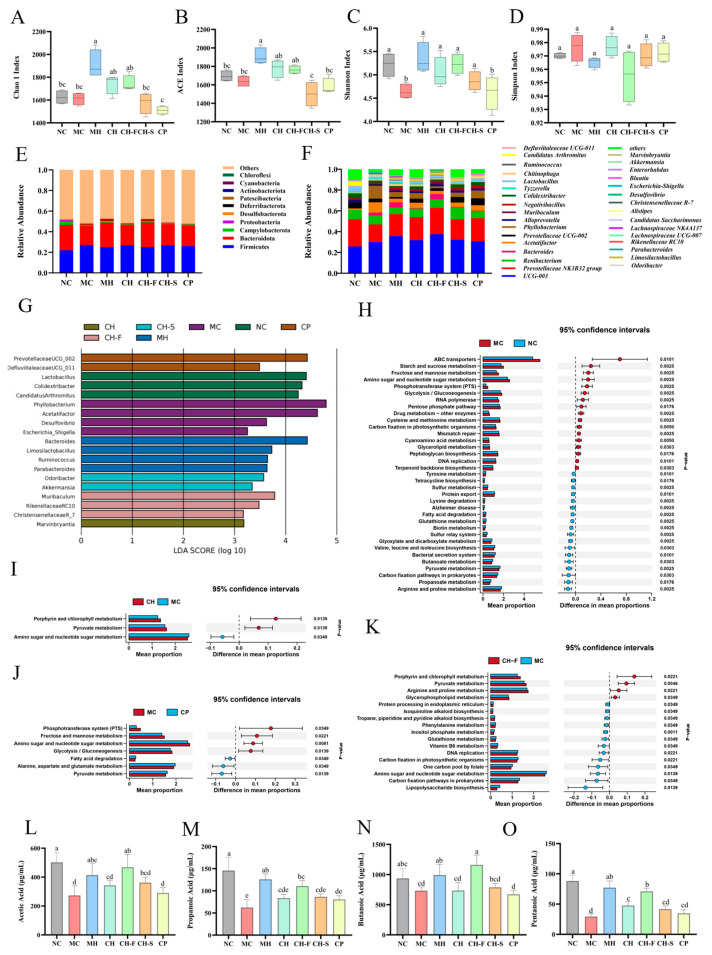
Effects of CH, CP, CH-F, and CH-S administration on gut microbiota and SCFAs (*n* = 6). The Chao1 (**A**), ACE (**B**), Shannon (**C**), and Simpson (**D**) α-diversity indices, as well as the phylum level (**E**) and genus level (**F**) compositions of the gut microbiota in each group. The LEfSe analysis (**G**) of the gut microbiota across all groups at the genus level. PICRUSt2 functional prediction analyses of the gut microbiota, comparing the MC group with the NC group (**H**), CH group (**I**), CP group (**J**), and CH-F group (**K**). Concentrations of acetic acid (**L**), propionic acid (**M**), butyric acid (**N**), and valeric acid (**O**) in cecal contents of mice from each group. For subfigures (**A**–**D**,**L**–**O**): data are presented as mean ± SD. One-way ANOVA followed by Tukey’s post-hoc test was used for group comparisons. Different lowercase letters indicate significant differences at *p* < 0.05, while the same letter represents no significant difference.

Four α-diversity indices, namely Chao1, ACE, Shannon, and Simpson, were employed to evaluate the richness and diversity of the gut microbiota in samples from each group (Figure 7A–D). The Chao1 and ACE indices in the MH, CH, and CH-F groups were significantly higher than those in the MC group. The Shannon index in the MC group was significantly lower than that in the NC group, and was significantly restored in the MH, CH, and CH-F groups. Figure 7E,F shows the gut microbiota composition at the phylum and genus levels in each group. At the phylum level, the Firmicutes/Bacteroidota (F/B) ratio in the MC group was significantly higher than that in the NC group (*p* < 0.05), whereas the F/B ratios in the MH, CH, and CH-F groups were significantly lower than that in the MC group (*p* < 0.05). Figure 7G presents the LEfSe analysis of the gut microbiota at the genus level among all groups (LDA > 2.0, *p* < 0.01). The NC group was enriched with *Lactobacillus, Colidextribacter* and *Candidatus Arthromitus*. The MC group was significantly enriched with *Desulfovibrio* and *Escherichia-Shigella*. The CH-F group was enriched with *Muribaculum*, *Rikenellaceae RC10* and *Christensenellaceae R-7*. The MH group was enriched with *Bacteroides*, *Limosilactobacillus* and *Ruminococcus*.

LEfSe analysis identified differential gut microbiota taxa among groups, and PICRUSt2 was used to predict the metabolic functions of the gut microbiota in each group. A total of 34 significantly differential KEGG metabolic pathways were identified between the NC and MC groups (Figure 7H–K). Among these, the MC group exhibited upregulation of pathways including starch and sucrose metabolism, glycolysis/gluconeogenesis, fructose and mannose metabolism, amino sugar and nucleotide sugar metabolism, and ABC transporters, whereas pathways such as butanoate metabolism, propanoate metabolism, and fatty acid degradation were downregulated. Compared with the MC group, the CH group showed only three significantly altered pathways: upregulation of pentose and glucuronate interconversion and phenylalanine metabolism, and downregulation of amino sugar and nucleotide sugar metabolism. Compared with the MC group, the CP group showed five significantly altered metabolic pathways, including upregulation of the phosphatidylinositol signaling system and amino sugar and nucleotide sugar metabolism, and downregulation of fatty acid degradation and alanine, aspartate and glutamate metabolism. The CH-F group showed 12 significantly differential pathways compared with the MC group, including upregulation of pentose and glucuronate interconversions, arginine and proline metabolism, and the bacterial secretion system, and downregulation of carbon fixation in photosynthetic organisms, fructose and mannose metabolism, and glycolysis/gluconeogenesis.

### 3.9. Effects of CP, CH, CH-F and CH-S on Cecal SCFA Levels

The acetic acid, propionic acid, butyric acid, and valeric acid levels in the cecal contents of mice from each group are presented in Figure 7L–O. The levels of all four SCFAs in the MC group were significantly lower than those in the NC group. In contrast, all treatment groups had increased cecal SCFA levels to varying degrees. Among all groups, the CH-F group showed the greatest elevation in acetic acid and butyric acid levels, increasing the respective levels by 70.97% (*p* < 0.001) and 58.41% (*p* < 0.001). Additionally, the CH-F group’s elevations in propionic acid and valeric acid levels were slightly lower than those of the MH group, with increases of 77.08% (*p* < 0.001) and 141.63% (*p* < 0.001), respectively.

### 3.10. Correlations of Gut Microbiota with SCFAs and Biochemical Indicators

**Figure 8:**
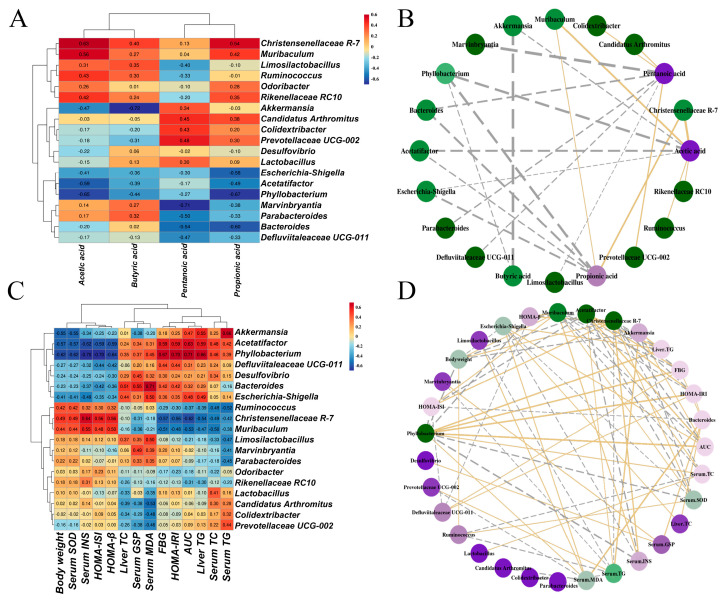
The heatmap of the Spearman’s correlation analysis between the gut microbiota and SCFAs (**A**), biochemical indexes (**C**), and their network plot (**B**,**D**) based on the correlation. In heatmaps (**A**,**C**), red squares indicate positive correlations, while blue squares indicate negative correlations. In network plots (**B**,**D**), yellow solid lines denote positive correlations, gray dashed lines denote negative correlations, and the width of lines is proportional to the strength of the correlation.

The Spearman correlation analysis was performed to evaluate the correlations between the differential bacterial genera in the gut microbiota and SCFAs, and network diagrams were plotted for data |r| > 0.4 and *p* < 0.05 (Figure 8A,B). Acetic acid was negatively correlated with *Acetatifactor*, *Phyllobacterium*, *Escherichia-Shigella* and *Akkermansia*, whereas it was positively correlated with *Muribaculum*, *Christensenellaceae R-7*, *Rikenellaceae RC10*, and *Ruminococcus*. Propionic acid showed a positive correlation with *Muribaculum* and *Christensenellaceae R-7*, *Candidatus Arthromitus* and *Rikenellaceae RC10*, but a negative correlation with *Phyllobacterium*, *Bacteroides*, *Acetatifactor*, and *Escherichia-Shigella*. Butyric acid was positively correlated with *Muribaculum*, *Christensenellaceae R-7*, *Rikenellaceae RC10*, *Limosilactobacillus* and *Ruminococcus*, while being negatively correlated with *Phyllobacterium*, *Acetatifactor* and *Akkermansia*. Pentanoic acid was positively correlated with *Colidextribacter*, *Candidatus Arthromitus*, and *Prevotellaceae UCG-002*, while being negatively correlated with *Marvinbryantia*, *Bacteroides*, *Parabacteroides*, *Defluviitaleaceae UCG-011*, and *Limosilactobacillus*.

The correlations and network plots between gut microbiota and biochemical indexes are shown in Figure 8C,D. *Akkermansia*, *Acetatifactor*, *Phyllobacterium*, *Defluviitaleaceae UCG-011*, *Desulfovibrio*, *Bacteroides*, and *Escherichia-Shigella* are negatively correlated with body weight, HOMA-β, HOMA-ISI, serum SOD, and serum INS, and positively correlated with serum MDA, serum TC, serum TG, serum GSP, AUC, liver TC, liver TG, FBG, and HOMA-IRI. Exceptions to this trend are observed for *Akkermansia*, which is negatively correlated with serum MDA, and *Bacteroides*, which is negatively correlated with serum TG. In contrast, *Ruminococcus*, *Christensenellaceae R-7*, and *Muribaculum* show a completely opposite correlation pattern. These genera are positively correlated with body weight, HOMA-β, HOMA-ISI, serum SOD, and serum INS, and negatively correlated with serum MDA, serum TC, serum TG, serum GSP, AUC, liver TC, liver TG, FBG, and HOMA-IRI. Additionally, *Limosilactobacillus*, *Marvinbryantia*, and *Parabacteroides* are positively correlated with liver TC, serum GSP, and serum SOD, and negatively correlated with serum TG. *Lactobacillus*, *Candidatus Arthromitus*, *Colidextribacter*, and *Prevotellaceae UCG-002* are negatively correlated with liver TC, serum GSP, and serum SOD, and positively correlated with serum TG and serum TC.

## 4. Discussion

As one of the most well-studied probiotic groups, *Lactobacillus* species exhibit both anti-diabetic and antioxidant effects, such as enhancing key antioxidant enzyme activity and lowering levels of lipid peroxidation [21]. Consistent with this, we found that all the strains used for screening showed good growth and high viability, with robust antioxidant and hypoglycemic enzyme inhibitory activities. Notably, we observed that the fermentation supernatant of all strains had significantly stronger antioxidant activity than the bacterial cells themselves, suggesting that the active antioxidant components may be mainly extracellular metabolites secreted by the strains [22]. In addition, we found that *L. rhamnosus* and *L. acidophilus* had the strongest α-glucosidase inhibitory activity, while *L. plantarumn* had excellent α-amylase inhibitory performance; inhibiting these two key carbohydrate-digesting enzymes can effectively delay postprandial blood glucose elevation [23]. Based on the screening results, we selected these three strains with complementary functions and favorable growth performance. Given the limited efficacy of single-strain probiotics in complex in vivo environments [24], we verified the optimal synergistic effect at a 1:1:1 ratio of the three strains, and further optimized the fermentation process via single-factor experiments combined with orthogonal array design.

Our fermentation of *C. pyrenoidosa* with the compound probiotics induced significant favorable changes in component composition, which aligns with well-documented beneficial biochemical transformations during food fermentation [25]. The reduced contents of total sugars, reducing sugars, and proteins in CH-F were probably due to the CPs utilizing carbohydrates as carbon sources for their growth and secreting proteases that hydrolyze macromolecular proteins into small-molecule peptides to meet their nutritional demands [26]. Meanwhile, the improved free radical scavenging capacity of CH-F was accompanied by a significant increase in polyphenol content; given that polyphenols are well-documented to possess strong reactive oxygen species-scavenging properties, this may partially account for the enhanced antioxidant activity [27]. The results preliminarily demonstrate that CH-F possessed superior in vitro hypoglycemic and antioxidant activities to CH, which thus prompted us to perform untargeted metabolomic analysis on CH and CH-F via UPLC-MS/MS. Among the main upregulated compounds, acetylcholine serves as a well-recognized potentiator of glucose-stimulated insulin secretion in normal β-cells [28], whereas citicoline has been demonstrated to exert protective effects against diabetic ocular complications [29]. In terms of the downregulated metabolites, they included various phosphatidylcholines (PCs) and one type of sphingomyelin (SM). PC and SM metabolism disorders are linked to diabetes, as they regulate ceramide accumulation, which inhibits insulin signaling via Akt/PKB pathway impairment, exacerbating insulin resistance and glucose intolerance [30]. These findings revealed the promising anti-diabetic potential of CH-F, prompting us to further investigate its anti-diabetic effects in animal experiments.

A high-sugar and high-fat diet combined with STZ represents a widely recognized and commonly adopted T2DM animal model, which well simulates the metabolic disturbance and pathological characteristics of T2DM [31]. Consistent with our in vitro findings, CH-F intervention exhibited the most prominent hypoglycemic efficacy among all self-prepared test agents in T2DM mice. Specifically, CH-F treatment effectively alleviated hyperglycemia, improved glucose tolerance, and restored insulin sensitivity and pancreatic β-cell function in T2DM mice, as reflected by the amelioration of HOMA-related indices [32]. Notably, CH-F outperformed the unfermented CH, sterilized CH-S and single CP interventions, confirming that fermentation significantly enhanced *C. pyrenoidosa*’s anti-diabetic potential. T2DM is closely associated with typical dyslipidemia, which is commonly characterized by systemic lipid metabolism disorder and excessive hepatic lipid accumulation that further exacerbates insulin resistance [33]. Previous studies have demonstrated that CH could effectively ameliorate the levels of TC and TG in diabetic rats [34], and our study further confirmed that CH fermented with CPs exerts a more potent effect on modulating dyslipidemia, which may be attributed to the synergism between live probiotics and fermentation-enriched bioactive substances in CH-F. Meanwhile, it mitigated hyperglycemia-induced oxidative stress by enhancing SOD activity and reducing MDA levels, a key driver of T2DM progression [35]. These biochemical improvements were further corroborated by histopathological observations: CH-F effectively alleviated hepatic steatosis and markedly restored the structural integrity of pancreatic islets, which provided direct histological evidence for its protective effect on metabolic organs and β-cell function [36].

Accumulating evidence indicates that the gut microbiota is a key factor in the pathophysiology of DM, and gut microbiota dysbiosis directly or indirectly contributes to diabetes by impacting intestinal barrier function and metabolic homeostasis [37]. Our results confirmed that CH-F intervention effectively ameliorated the gut dysbiosis in T2DM mice: it restored the reduced microbial diversity, partially restored the elevated F/B ratio that is closely linked to insulin resistance [38], and suppressed the enrichment of harmful taxa such as *Desulfovibrio* and *Escherichia-Shigella* [39,40]. Additionally, CH-F treatment selectively enriched a panel of beneficial SCFA-producing bacteria, including *Muribaculum*, *Christensenellaceae R-7* and *Rikenellaceae RC10* [41,42]. PICRUSt2 functional prediction analysis further revealed that, compared with CH and CP intervention, CH-F exerted a more robust regulatory effect on gut microbial metabolic pathways, further validating that fermentation significantly enhanced *C. pyrenoidosa*’s functional potential.

As the primary bioactive metabolites generated by gut microbiota fermentation of dietary fibers, SCFAs play a critical role in maintaining glucose homeostasis, improving insulin sensitivity and alleviating metabolic inflammation [43]. Consistent with the enrichment of SCFA-producing bacteria, we found that CH-F intervention significantly elevated the levels of all four major SCFAs in the intestinal tract of T2DM mice, with the most prominent improvement in acetic acid and butyric acid levels, which are the key mediators of the anti-diabetic effects of gut microbiota [44].

To further investigate the correlations between the altered gut microbiota, key biochemical indicators, and SCFAs, Spearman correlation analysis was performed. The results confirmed that the SCFA-producing bacteria enriched by CH-F were significantly positively correlated with both SCFA levels and the improved biochemical indicators including insulin sensitivity, β-cell function and lipid profiles. The harmful taxa depleted by CH-F showed the opposite correlation trend. Notably, these key functional taxa, such as *Christensenellaceae R-7* and *Muribaculum*, have been widely reported to be negatively associated with insulin resistance and T2DM progression [45], while the depleted harmful taxa like *Desulfovibrio* have been linked to hepatic lipid damage and inflammation [46]. In light of the results above, we hypothesized that CH-F alleviated diabetic symptoms in mice by regulating the gut microbiota, among which *Christensenellaceae R-7* and *Muribaculum* may be the potential key bacterial taxa involved in this process, though the causal mediation effect needs to be further verified by follow-up experiments.

While our findings demonstrate that CH-F exerts favorable anti-diabetic effects in preclinical models, several important limitations of this work must be noted. First, although the STZ-induced T2DM mouse model is widely used for preclinical evaluation of anti-diabetic agents, interspecies differences between mice and humans pose substantial challenges for the translational relevance of our results. Additionally, although Spearman analysis further revealed the correlations between gut microbiota, biochemical indices, and SCFAs, it should be noted that such correlations did not imply a causal relationship. The underlying mechanisms of CH-F’s ameliorative effects on T2DM remain to be clarified, and will be further investigated in our future studies. Moreover, to translate the promising anti-diabetic potential of CH-F into a functional food ingredient, maintaining the stability of its liquid bioactive metabolites during long-term storage and processing remains a practical challenge. Future research could explore advanced formulation technologies, such as microencapsulation using composite wall materials like cellulose nanocrystals and gelatin [47], to enhance the physicochemical stability and targeted gastrointestinal delivery of CH-F formulations.

## 5. Conclusions

This study developed a compound probiotics-fermented *C. pyrenoidosa* product (CH-F) with enhanced anti-diabetic activity and explored its underlying mechanism. The results demonstrated that CH-F showed altered nutritional composition and superior in vitro hypoglycemic and antioxidant activities compared with unfermented CH. Untargeted metabolomics revealed that CH-F was enriched in acetylcholine, citicoline and other potential anti-diabetic metabolites, laying the chemical foundation for its enhanced efficacy. In STZ-induced T2DM mice, CH-F effectively alleviated the core pathological features of T2DM, including hyperglycemia, dyslipidemia and oxidative stress, with an overall efficacy significantly superior to unfermented CH. Meanwhile, CH-F markedly mitigated the histological injuries of the liver and pancreas in diabetic mice, and effectively restored impaired insulin sensitivity and pancreatic β-cell function. Furthermore, CH-F effectively regulated the gut microbiota structure in T2DM mice, partially reversed the elevated F/B ratio, and enriched the abundance of key beneficial bacteria, including *Muribaculum* and *Christensenellaceae R-7*. Consistent with the regulatory effect on gut microbiota, CH-F also significantly increased the concentrations of SCFAs in the cecal contents of T2DM mice. Correlation analysis indicated that CH-F might exert its anti-diabetic effects by increasing the abundance of SCFA-producing bacteria. These findings highlight the promising anti-diabetic potential of CH-F as a functional food, though further clinical validation remains warranted given the preclinical nature of this study.

## Figures and Tables

**Table 1:** Design of compound probiotics for fermentation.

Group	Culture Type	Strain Composition
1	Monoculture	*L. rhamnosus* (single strain)
2	Monoculture	*L. plantarum* (single strain)
3	Monoculture	*L. acidophilus* (single strain)
F-1	Dual co-culture	*L. rhamnosus*:*L. plantarum* = 1:1
F-2	Dual co-culture	*L. rhamnosus*:*L. acidophilus* = 1:1
F-3	Triple co-culture	*L. rhamnosus*:*L. plantarum*:*L. acidophilus* = 1:1:1
F-4	Triple co-culture	*L. rhamnosus*:*L. plantarum*:*L. acidophilus* = 2:1:1

**Table 2:** Factors and levels of the orthogonal experiment.

Level	A (*C. pyrenoidosa* Content/%)	B (Glucose Content/%)	C (Temperature/°C)	D (Time/h)
1	4	3	34	24
2	5	4	37	30
3	6	5	40	36

**Table 3:** Design and results of the orthogonal experiment.

No	A (%)	B (%)	C (°C)	D (h)	α-Glucosidase Inhibition (i%)	α-Amylase Inhibition (j%)	pH (p)	CFU (c)	Z-Score
1	1	1	1	1	32.92%	50.88%	4.06	9.32	−1.413
2	1	2	2	2	28.24%	59.27%	3.98	9.21	−2.473
3	1	3	3	3	32.74%	78.75%	3.91	9.30	0.375
4	2	1	2	3	23.98%	51.39%	3.93	9.15	−3.962
5	2	2	3	1	39.90%	62.76%	4.05	9.33	0.949
6	2	3	1	2	42.09%	94.37%	4.06	9.35	2.713
7	3	1	3	2	37.48%	27.74%	4.04	9.48	−0.348
8	3	2	1	3	43.57%	60.00%	3.93	9.26	3.172
9	3	3	2	1	39.80%	62.91%	4.10	9.40	0.922
K_i1_	93.90	94.38	118.58	112.52	A_1_B_3_C_1_D_1_	A > C > B > D			
K_i2_	105.87	111.61	92.02	107.81					
K_i3_	120.85	124.63	110.02	107.81					
R_i_	26.95	20.25	26.56	4.71					
K_j1_	188.90	165.01	205.25	176.55	A_2_B_3_C_1_D_2_	B > D > C > A			
K_j2_	208.52	182.03	173.57	216.38					
K_j3_	185.65	236.03	204.25	190.14					
R_j_	22.87	71.02	31.68	39.83					
K_p1_	11.95	12.03	11.22	12.22	A_2_B_3_C_3_D_1_	D > B > C > A			
K_p2_	12.02	11.17	12.01	12.05					
K_p3_	11.27	12.04	12.01	10.97					
R_p_	0.75	0.87	0.79	1.25					
K_c1_	27.83	12.03	11.22	12.22	A_3_B_3_C_1_D_2_	C > B > D > A			
K_c2_	27.89	27.86	27.76	28.04					
K_c3_	28.14	28.05	28.17	27.71					
R_c_	0.31	16.02	16.95	15.82					

Note: A, B, C, and D represent the four factors of the orthogonal experiment, which correspond to *C. pyrenoidosa* content (%), glucose content (%), temperature (°C), and time (h), respectively.

**Table 4:** ANOVA of the orthogonal experiment.

	Source of Variation	SS	df	MS	F Value	*p* Value
α-glucosidase Inhibition (%)	A	574.968	2	287.484	147.155	<0.01
	B	242.207	2	121.104	61.990	<0.01
	C	105.613	2	52.806	27.030	<0.01
	D	138.49	2	69.245	35.445	<0.01
α-amylase Inhibition (%)	A	1732.589	2	866.295	164.532	<0.01
	B	5619.542	2	2809.7701	533.648	<0.01
	C	772.79	2	386.395	73.386	<0.01
	D	94.857	2	47.428	9.009	<0.05
pH	A	0.334	2	0.167	2503.500	<0.01
	B	0.499	2	0.249	3741.500	<0.01
	C	0.406	2	0.203	3042.000	<0.01
	D	0.904	2	0.452	6778.500	<0.01
CFU	A	0.045	2	0.023	95.813	<0.01
	B	0.016	2	0.008	32.953	<0.01
	C	0.077	2	0.039	163.312	<0.01
	D	0.079	2	0.039	166.359	<0.01

**Table 5:** The main differential metabolites between CH and CH-F.

Name	Formula	Classification	Up (↑)/ Down (↓)
dUMP	C_9_H_13_N_2_O_8_P	Deoxyribonucleotides	↑
Acetylcholine	C_7_H_15_NO_2_	Neurotransmitters	↑
Estrone sulfate	C_18_H_22_O_5_S	Estrogens	↑
Citicoline	C_14_H_26_N_4_O_11_P_2_	Nucleoside diphosphocholines	↑
Linolelaidic acid	C_18_H_32_O	Unsaturated fatty acids	↑
PC 15:0_16:2	C_39_H_74_NO_8_P	Glycerophosphocholines	↓
SM 8:1; 2O/32:7	C_45_H_77_N_2_O_6_P	Sphingomyelins	↓
PC 16:1_16:1	C_40_H_76_NO_8_P	Glycerophosphocholines	↓
MGDG	C_43_H_74_O_10_	Glycosylglycerides	↓
PC 16:2_18:2	C_40_H_72_NO_8_P	Glycerophosphocholines	↓
Methyladenosine	C_11_H_15_N_5_O_4_	Purine nucleosides	↓
PC 16:0_18:5	C_42_H_74_NO_8_P	Glycerophosphocholines	↓

## Data Availability

The original contributions presented in the study are included in the article/Supplementary Material; further inquiries can be directed to the corresponding authors.

## Supplementary Materials

The following supporting information can be downloaded at: https://www.mdpi.com/article/10.3390/foods15101739/s1. Figure S1: Total ion chromatography of CH (A) and CH-F (B) in positive and negative ion modes by UPLC-MS/MS.

## Abbreviations

The following abbreviations are used in this manuscript:

ABTS	2,2′-Azinobis-(3-ethylbenzothiazoline-6-sulfonic acid)
ANOVA	One-way analysis of variance
CFU	Colony forming unit
CH	*Chlorella pyrenoidosa* solution
CHM	*Chlorella pyrenoidosa* medium
CH-F	Fermented *Chlorella pyrenoidosa* product
CH-S	Sterilized fermented *Chlorella pyrenoidosa* product
CP	Compound probiotic
DM	Diabetes mellitus
DNS	Dinitrosalicylic acid
DPPH	2,2-Diphenyl-1-picrylhydrazyl
F/B	Firmicutes/Bacteroidota
FBG	Fasting blood glucose
FC	Fold change
GSP	Glycosylated serum protein
HOMA-IRI	Insulin resistance index
HOMA-ISI	Insulin sensitivity index
HOMA-β	Pancreatic β-cell function index
INS	Serum insulin
LEfSe	Linear discriminant analysis effect size
MDA	Malondialdehyde
MRS	De Man, Rogosa and Sharpe
OD	Optical density
OGTT	Oral glucose tolerance test
OH	Hydroxyl radical
PC	Phosphatidylcholine
SCFAs	Short-chain fatty acids
SOD	Superoxide dismutase
SM	Sphingomyelin
STZ	Streptozotocin
TC	Total cholesterol
TCA	Trichloroacetic acid
TG	Triglyceride
T2DM	Type 2 diabetes mellitus
