# Supplementary material for: Probiotic Fermentation Enhances Anti-Diabetic Effects of Chlorella pyrenoidosa in Mice by Modulating Gut Microbiota and Short-Chain Fatty Acids
Source: Foods. 2026 May 14;15(10):1739. doi: 10.3390/foods15101739 (PMC13205893; doi:10.3390/foods15101739)
Supplement: Supplementary file 1 [file foods-15-01739-s001.zip › foods-4268580-supplementary.pdf]

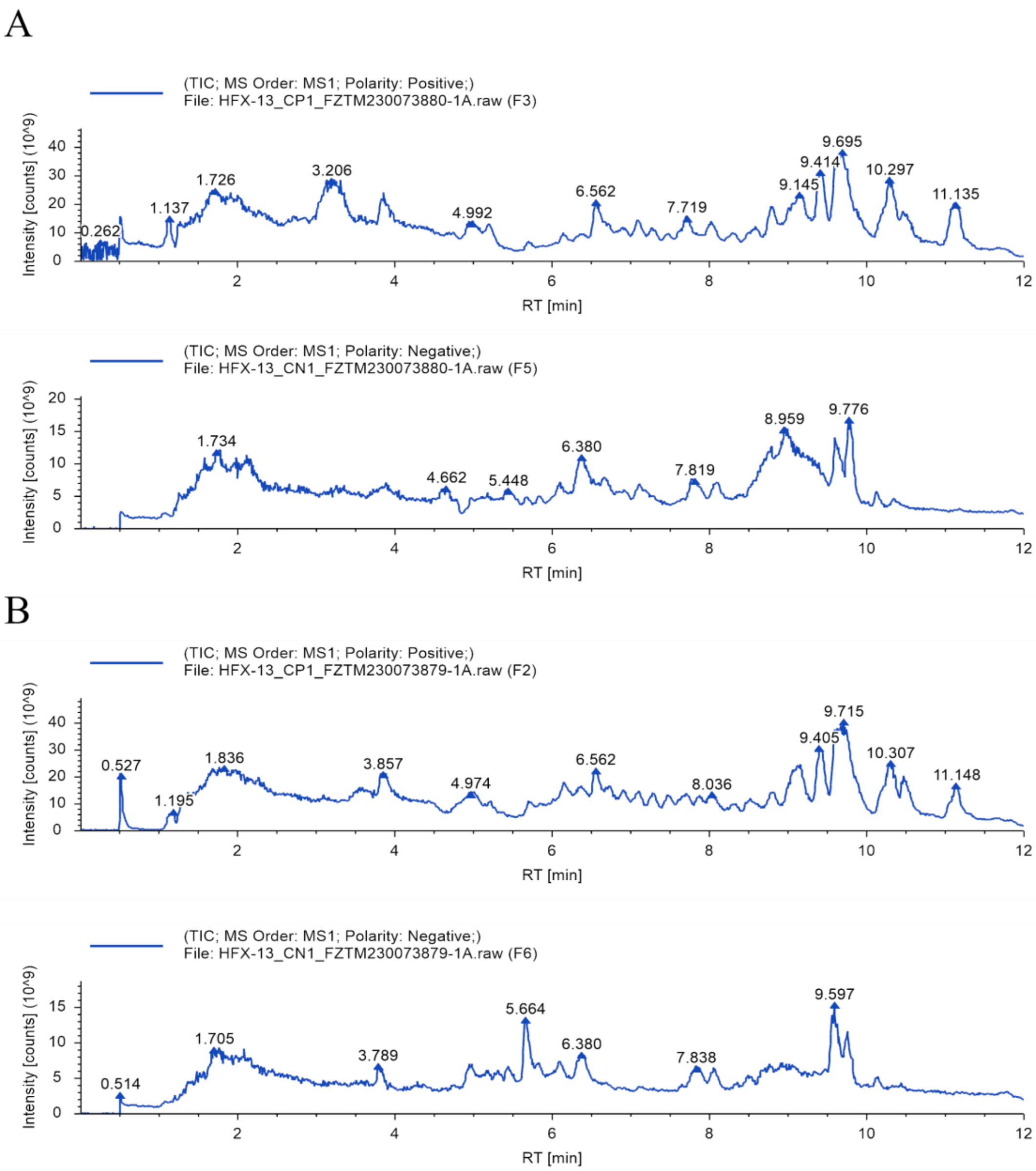

Figure S1. Total ion chromatography of CH (A) and CH-F (B) in positive and negative ion modes by UPLC-MS/MS.
